# Review of Osteoporotic Fractures: Occurrence, Prevention, and Consequences

**DOI:** 10.1055/s-0044-1789220

**Published:** 2025-06-14

**Authors:** Nelson Elias, José Eduardo Grandi Ribeiro, Luiz Augusto Campinho, Cilas Reis, Luiz Arthur Miguelote S. Elias, Pedro José Labronici

**Affiliations:** 1 Universidade Estadual Rio de Janeiro, Rio de Janeiro, RJ, Brazil; 2 Universidade Federal Rio de Janeiro, Rio de Janeiro, RJ, Brazil; 3 School of Medical Sciences, Santa Casa de Misericórdia de Vitória (EMESCAM), Vitória, ES, Brazil; 4 Orthopedics and Traumatology Service, Hospital Estadual de Urgência e Emergência de Vitória (HEUE), Vitória, ES, Brazil; 5 School of Medicine, Universidade Vila Velha, Vila Velha, ES, Brazil; 6 Universidade Federal Fluminense (UFF), Niterói, RJ, Brazil

**Keywords:** diagnosis, fractures, stress, osteoporosis, risk assessment, treatment, avaliação de risco, diagnóstico, fraturas por estresse, osteoporose, tratamento

## Abstract

Osteoporosis is a metabolic condition that compromises bone density and architecture, increasing the risk of fractures and impacting morbidity and mortality. The diagnosis involves bone densitometry, in which mineral density in areas prone to fractures is assessed. Primary osteoporosis is age-related and may remain asymptomatic for years, while secondary osteoporosis results from comorbidities or medications. Approximately 80% of postmenopausal Caucasian women have osteoporosis, with an expected increase with aging. Orthopedic treatments are common for fractures, which are often caused by falls in the elderly. Fracture prevention requires public health policies and therapies focused on this goal.

## Resumo

A osteoporose é uma condição metabólica que compromete a densidade e a arquitetura ósseas, o que aumenta o risco de fraturas e tem impacto na morbimortalidade. O diagnóstico envolve a densitometria óssea, em que se avalia a densidade mineral em áreas propensas a fraturas. A osteoporose primária, relacionada à idade, pode permanecer assintomática por anos, ao passo que a secundária resulta de comorbidades ou medicamentos. Cerca de 80% das mulheres brancas na pós-menopausa têm osteoporose, com expectativa de aumento com o envelhecimento. Tratamentos ortopédicos são comuns para fraturas, que são frequentemente causadas por quedas em idosos. A prevenção de fraturas é crucial, e requer políticas de saúde pública e terapias com este objetivo.

## Introduction

### Definition

Osteoporosis is a decrease in bone density and quality, which increases the risk of fractures due to bone porosity and fragility. Bone loss is gradual and asymptomatic; as such, osteoporosis is often called a “silent disease”. Fractures, usually in the spine, wrist, hip, and shoulder, are the first obvious symptoms. 1 2

### Epidemiology

Osteoporosis is a severe global public health problem, affecting approximately 200 million women worldwide and with an increasing incidence in older age groups. 1 2 3 4 Approximately 30% of postmenopausal women in the United States and Europe have osteoporosis, and a significant proportion will suffer fragility fractures throughout their lives. 2 Populational aging will contribute to a substantial increase in osteoporosis incidence, and projections indicate significant increases in hip fracture rates by 2050 compared with 1990. 3

### Healthcare Costs

Osteoporosis impacts more than 10 million adults in the United States. It causes high social costs (22 billion dollars in 2008) and is underdiagnosed and undertreated. Projections indicate an increase in the annual number of fractures from 1.9 million to 3.2 million by 2040, with associated costs exceeding US$95 billion. Osteoporosis-related interventions can reduce fractures and expenses. 4 5 6 7 8 The economic burden exceeds that of conditions such as migraine and resembles that of rheumatoid arthritis. Continued use of osteoporosis medications reduces fracture risks and healthcare costs. Predictions show direct medical costs reaching $25.3 billion by 2025, highlighting the importance of improving medication persistence for payers and patients. 4 5

### Risk Factors

The risk factors for the development of osteoporosis can be permanent (such as age and gender) and modifiable (including smoking, alcohol consumption, and eating habits). These factors increase the likelihood of osteoporosis development, and the presence of multiple factors elevates the risk. Presenting risk factors does not necessarily lead to the development of osteoporosis; however, the greater the number and intensity of risk factors, the higher the probability of developing the conditionis. 2 5 6 7 8 9

### History of Osteoporosis Research

Osteoporosis, a condition affecting the bones, was identified approximately three centuries ago, although the disease has existed for millennia. The discovery of the bone-remodeling process by John Hunter 250 years ago was crucial for the understanding of bone tissue removal and replacement. 10 Surgeon Astley Cooper linked the decline in bone density to an increased risk of fractures. Jean Lobstein introduced the term *osteoporosis* in the 1830s. 11 12

At the beginning of the twentieth century, Kyes and Potter 13 observed the relationship between estrogen levels and bone density in pigeons. Later, Fuller Albright et al. 14 15 contributed significantly to the understanding of osteoporosis by identifying osteoblast deficiency and associating it with menopause in women. They developed the first effective osteoporosis treatment using estrogen.

In 1955, Alexander Cooke 16 defined osteoporosis as an inadequate bone formation due to a lack of “matrix”, suggesting that histological examinations should base the diagnosis. Cooke 16 also mentioned the beneficial and adverse effects of androgens. Subsequent therapeutic trials with fluoride, anabolic steroids, and calcitonin have had limited success and some side effects. 15 16 17 18

## Pathogenesis

Osteoporosis can occur due to failure to reach peak bone mass, excessive bone resorption, decreased bone formation during remodeling, or any combination of these factors. They all likely contribute to osteoporosis in varying degrees. 19

### Bone Mass Peak

Peak bone mass occurs when bone mineral density (BMD) reaches its highest point. Bone mass increases during childhood and adolescence, reaching its peak around age 30. After that, BMD gradually decreases with aging. Reducing this loss is crucial to prevent osteoporosis and fractures in adulthood. A 10% increase in peak bone mass can result in a 30% reduction in hip fractures. Genetic factors play a significant role, contributing to about 80% of the variability in peak bone mass, as evidenced by twin studies. Genetic variants, including low-density lipoprotein receptor-related protein 5 (LRP5) 5 and sclerostin, have been identified by genome-wide association studies. 19 Moreover, environmental factors, such as nutrition, exercise, and smoking, play key roles in bone mass peak. 19 Modulation of this peak can occur during intrauterine life, being affected by maternal nutrition, smoking, and exercise levels. 20

### Imbalance in Bone Resorption and Formation

Bone remodeling involves a coordinated action of osteoclasts and osteoblasts and is crucial to maintain bone health in adult life and repair microdamage. Although increased bone resorption appears to influence bone loss and the risk of fracture, compromised bone formation also plays a critical role in osteoporosis. 21 This impairment partly results from a reduced number of osteoprogenitor/preosteoblastic cells, an age-related defect in transforming stromal cells into adipocytes rather than osteoblasts, or both. Age- or menopause-associated bone loss is a significant osteoporosis determinant, and genetic factors account for variations in skeletal integrity among elderly people in the same age group. 22 23

### Osteoporosis-related Conditions

Subjects with certain health conditions resulting in higher bone loss and/or risk of falls present a greater risk of developing osteoporosis. 24 25 Secondary osteoporosis results from several comorbidities, medications, or both ( Table 1 ), e and it is associated with diseases that affect the mechanisms involving the balance of calcium, vitamin D, and sexual hormones. 26 About a third of postmenopausal women, in addition to many men and premenopausal females, present concomitant causes of bone loss, 25 such as renal hypercalciuria, which is treatable with thiazide diuretics. 26 Treatment is specific for each associated disease and requires a multidisciplinary approach. 25

**Table 1:** Causes of secondary osteoporosis in adults 25

Rheumatoid arthritis and other rheumatological conditions
Malabsorption syndromes
Sex hormone deficiency
Primary hyperparathyroidism
Chronic kidney disease
Chronic liver disease
Diabetes
Chronic obstructive pulmonary disease
Untreated hyperthyroidism
Neurological conditions
Cancer

### Metabolism and Bone Regulation in Osteoporosis Patients

Bones undergo constant remodeling through a balance between their formation by osteoblasts and resorption by osteoclasts. 9 Osteoporosis increases the risk of fracture due to reduced bone density. 16 17 21 Metabolic bone disease encompasses abnormalities from genetic disorders or mineral (calcium and phosphorus) and vitamin D deficiencies, including osteoporosis, osteomalacia, rickets, and bone Paget disease. 27

Osteoporosis may result from inadequate calcium intake and vitamin D deficiency, 28 and proper vitamin D levels help maintain bone strength. Despite this, calcium and vitamin D therapy may not completely prevent bone loss, and although estrogen- and progesterone-based hormone therapy slows osteoporosis progression, it has side effects. Bone metabolism tests may offer therapeutic alternatives.

Blood levels determine the supplementation doses of calcium and vitamin D. certain indicators, such as urinary calcium levels and hormonal tests, help assess intestinal absorption and determine the origin of osteoporosis (primary or secondary). 28

## Calcium and Phosphate in Bone Metabolism

Bone and blood calcium levels are essential for physiological balance. They are dynamic and regulated by hormones, including vitamin D, calcitonin, and the parathyroid hormone (PTH). Vitamin D facilitates intestinal calcium absorption, maintaining adequate levels for bone mineralization and hypocalcemic tetany prevention. Vitamin D deficiency can lead to brittle and deformed bones, while proper vitamin D levels prevent rickets in children, osteomalacia in adults, and osteoporosis in the elderly. 28 29 30 31

Osteoporosis deteriorates bone microarchitecture. Its assessment may use bone turnover markers (BTMs) to complement BMD determination and evaluate the risk of fracture. Sex hormones, especially estrogen, play a crucial role in primary osteoporosis, and estrogen deficiency in menopause is a significant cause of osteoporosis. A lack of estrogen directly affects osteoclasts, leading to bone disorders, including osteoporosis. 32 33

### Assessment of Bone Mineral Density

**Figs. 1 and 2:**
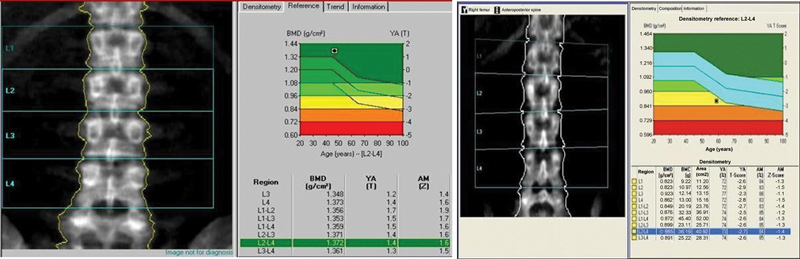
Bone mineral density (BMD) values of a healthy elderly woman ( Fig. 1 ) and an elderly woman with osteoporosis ( Fig. 2 ). Fig. 1 shows a BMD test of a healthy elderly woman. Radiography reveals normal hip bones. The graph shows the BMD value within the green (normal) zone. Fig. 2 shows a BMD test from an elderly woman with osteoporosis. Radiography shows weaker hip bones. The graph shows the BMD value within the red (osteoporotic) zone, significantly increasing the risk of fractures. **Abbreviation:** BMC, bone mineral content.

The Z-score on bone densitometry indicates the number of standard deviations above or below the expected value from a subject with the same age, gender, weight, and ethnic or racial origin as those of the patient under evaluation. A low Z-score suggests abnormal bone loss, and a high score is normal. This score is not age-related and may indicate an underlying, treatable condition to slow or stop bone loss 34 35 36 37 ( Figs. 1,2 ).

Radiography is a non-invasive medical test widely used to diagnose and treat medical conditions. It is the oldest imaging method and can examine the entire body or specific body parts using low doses of ionizing radiation. In some cases, peripheral radiography or ultrasound devices may evaluate low bone mass. Computed tomography (CT) with special software can also diagnose or monitor low bone mass; however, it is used less frequently than LRP screening through dual-energy X-ray absorptiometry (DXA). 36 37

### Fracture Types

Osteoporosis is an asymptomatic condition frequently diagnosed after fractures, which are the main manifestation of the disease and evidence bone atrophy. Age-related osteoporosis is underdiagnosed because it remains silent for several years until fractures occur, especially in the elderly, resulting in daily activity limitation. Osteoporotic fractures can result in severe complications, increasing morbidity and mortality rates. 9 10 Adult patients with fractures must undergo osteoporosis screening, since approximately 30% of the cases have secondary causes, notably in premenopausal women, men with osteoporosis, and patients with hip fractures. Appropriate laboratory tests are useful to investigate secondary causes. 3 9 10 11

The increasing incidence of fractures in the elderly has become a significant health problem in several countries. Moreover, these issues will probably increase due to populational aging. More than 90% of fractures result from low-energy falls and cause high mortality rates. For instance, the incidence of hip fractures in Australia is expected to increase from 20 thousand to 50 thousand cases in 2050 due to the aging of the population. 8 9 11 12

**Figs. 3 A,B and 4 A,B:**
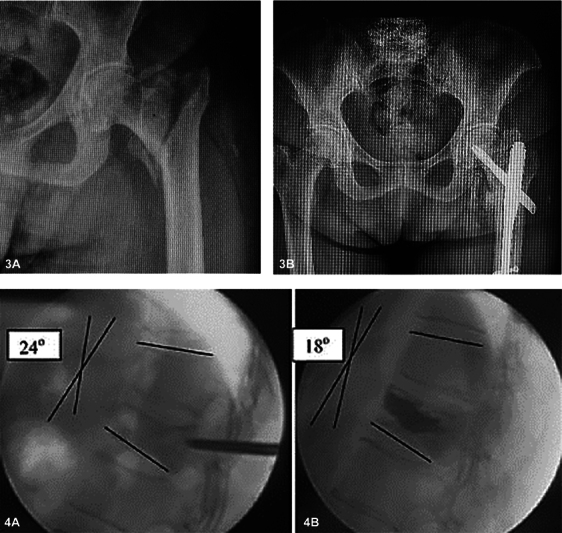
Examples of the two most common sites affected by osteoporotic fractures and their orthopedic treatments. ( **3A** ) Preoperative transtrochanteric fracture. ( **3B** ) Surgical treatment. ( **4A** ) Preoperative assessment of vertebral fracture. ( **4B** ) Surgical treatment after kyphoplasty (intraosseous injection of orthopedic cement). Angular kyphosis was reduced from 24 to 18 degrees.

Osteoporotic fractures, often resulting from falls, mainly affect the vertebrae, hips, wrists, and shoulders ( Figs. 3,4 ). Osteoporosis affects approximately 80% of postmenopausal Caucasian women and will have an increasing impact as the population ages. 11 12 13 These fractures, especially of the hip and vertebra, have a 12-month mortality rate of up to 20% due to hospitalization and increased risk of complications. Diagnosis often occurs after the first fracture, usually requiring orthopedic surgical treatment. 4 5 11

The validated Fracture Risk Assessment Tool (FRAX) calculates the 10-year fracture risk based on individual factors, including BMD. The FRAX is widely integrated into national guidelines, affordable, easy to apply by healthcare professionals, and useful, considering the exponential increase in hip fracture rates with age. 4 11 12 38

### Identification and Determination of the Risk of Fracture

Osteoporosis increases the risk of fractures due to low BMD, impaired bone microarchitecture/mineralization, reduced bone strength, or any combination of these factors. Often asymptomatic, it is frequently diagnosed following low-trauma fractures of the hip, spine, proximal humerus, pelvis, or wrist, which may require hospitalization, representing a significant economic burden for healthcare systems. The risk factors include permanent features, such as age and gender, and modifiable ones, related to personal lifestyle. 4 5 6 7 8 9

### Permanent Risks

Permanent risk factors such as age, female gender, family history of osteoporosis, previous fracture, ethnicity, menopause/hysterectomy, long-term glucocorticoid therapy, rheumatoid arthritis, and primary/secondary hypogonadism in men cannot undergo any change. However, subjects need to be aware of these factors to take steps to help reduce bone mineral loss. Additionally, secondary risk factors from disorders and medications weaken bones, increasing the risk of fall-related fractures. 2 5 6

### Modifiable Risks

Modifiable risk factors such as alcohol consumption, smoking, low body mass index, poor nutrition, vitamin D deficiency, eating disorders, lack of exercise, low dietary calcium intake, and frequent falls directly affect BMD and increase the risk of fractures. 7 8 The diagnosis of osteoporosis often relies on BMD measurements, especially in the hip and lumbar spine, using the DXA device. The FRAX, which considers factors such as age, race, alcohol consumption, gender, body mass index, smoking history, fracture history, and BMD, is an effective tool to predict the 10-year risk of osteoporotic fractures.

## Treatment

a) Medical drug therapy evidence.
b) Non-pharmacological management and physical activity medical drug therapy evidence.

Osteoporosis treatment depends on its cause. Treatment of secondary osteoporosis is more complex, as it directly relates to the underlying disease. Pharmacological therapy aims to reduce the risk of fractures, and many therapeutic methods also prevent osteoporosis. 1 2

Primary osteoporosis, often linked to age and sex hormone deficiency, results in continued deterioration of bone trabeculae. In age-related osteoporosis, reduced estrogen production in postmenopausal women leads to significant bone loss. Sex hormone-binding globulin, which inactivates testosterone and estrogen in men during aging, may contribute to the decreased BMD over time. 37 38 39

Secondary osteoporosis results from several comorbidities, medications, or both. These comorbidities affect mechanisms leading to calcium, vitamin D, and sexual hormone imbalances. For example, in Cushing syndrome, the excess production of glucocorticoids can accelerate bone loss. Glucocorticoids are the medications most commonly involved in drug-induced osteoporosis, with a rapid bone level reduction within 3 to 6 months of starting the therapy. 39 40

Secondary osteoporosis has different causes depending on the gender of the subject. In male patients, excessive alcohol intake, the use of glucocorticoids, and hypogonadism are more common. Among female subjects, 32.4% of the cases have secondary causes, especially hypercalciuria, calcium malabsorption, hyperparathyroidism, vitamin D deficiency, hyperthyroidism, Cushing disease, and hypercalcemia, according to Tannenbaum et al. 41 Doctors and patients should talk about the best treatment based on the patient's needs and preferences and address the advantages and disadvantages of treatment alternatives. If patients are unable to understand the information, their caregivers should participate in the discussion, according to the guidelines of the United Kingdom's National Institute for Health and Care Excellence (NICE). 42

Osteoporosis medications have different mechanisms of action to strengthen bones and reduce the risk of fractures. Bisphosphonates inhibit osteoclasts for BMD preservation. Selective estrogen receptor modulators (SERMs) such as raloxifene act as agonists in some bone tissues. Hormone-replacement therapy (HRT) uses estrogen and progesterone in postmenopausal women. Receptor activator of nuclear factor Kappa-B ligand (RANKL) inhibitors, such as denosumab, reduce bone resorption. Teriparatide is a PTH analog that stimulates bone formation. Medications such as romosozumab stimulate bone formation by inhibiting sclerostin, an osteocyte-produced protein. Additionally, calcium and vitamin D are often recommended to maintain bone health. Treatment selection relies on individual factors under the guidance of a specialized physician.

The first-line treatment for postmenopausal women is bisphosphonates, whether alendronate or risedronate. Intravenous bisphosphonates or denosumab are recommended for patients who cannot tolerate oral bisphosphonates, and HRT with raloxifene or teriparatide may also be considered. 42

**Fig. 5:**
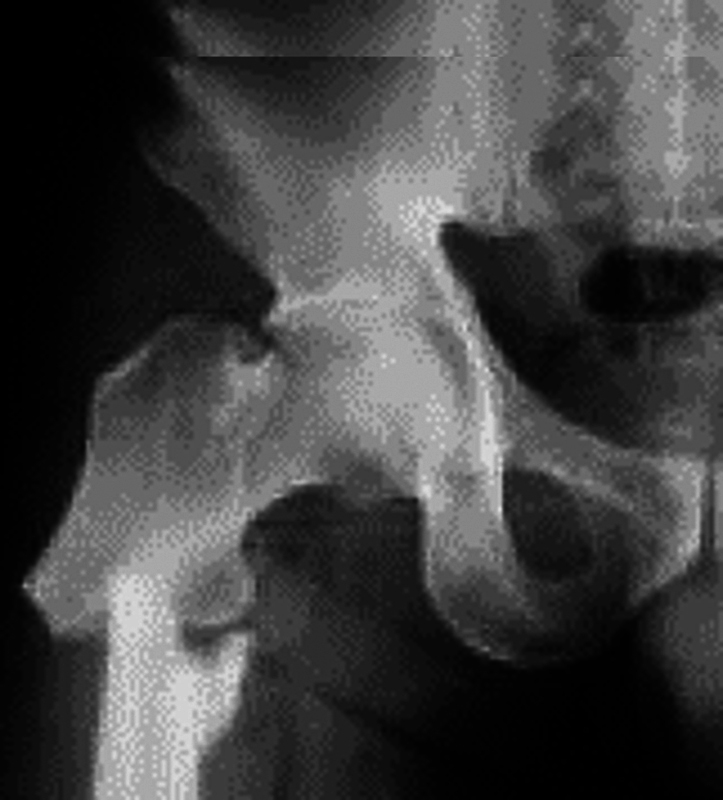
Radiograph showing an atypical right subtrochanteric femoral fracture in an elderly person after minimal trauma.

An atypical femoral fracture can occur after minimal trauma, especially in subjects under chronic bisphosphonate therapy ( Fig. 5 ). The recommendation is to stop these medications, consider treatment with teriparatide, and evaluate vitamin D and calcium supplementation. Prolonged bisphosphonate use impairs bone quality by inhibiting cellular bone remodeling. The contralateral side requires examination to determine if the condition is bilateral. 43 Despite the care currently provided, the prognosis for these fractures remains unfavorable, with a consolidation time ranging from 12 to 60 months. 43 Alendronate and risedronate are first-line treatments for men, with zoledronic acid, denosumab, or teriparatide as alternatives. Femoral neck BMD T-scores in men should be based on the United States' National Health and Nutrition Examination Survey (NHANES) female reference database. 44

Bone protection therapy is recommended for subjects older than 70 years of age under high doses of glucocorticoids, especially prednisolone at does higher than 7.5 mg/day. Postmenopausal women and younger men under high doses of glucocorticoid should also consider this therapy. For those at high risk of fractures, bone protection therapy should start at the beginning of the glucocorticoid regimen, with alendronate and risedronate as first-line treatments. 44

### Selecting Guidelines and Recommendations

The systematic review by Solomon et al. 45 highlights the consistency in osteoporosis guidelines, specifically those from the American Association of Clinical Endocrinologists (AACE) and the American College of Endocrinology (ACE). 24 To manage postmenopausal osteoporosis, the first-line treatment options include alendronate, risedronate, zoledronic acid, or denosumab for patients with no previous fragility fractures or at moderate fracture risk. Denosumab, teriparatide, or zoledronic acid are first-line treatments for patients with previous fragility fractures or high fracture risk. Alendronate and risedronate are treatment options. The American College of Physicians (ACP) 37 suggests using alendronate, risedronate, zoledronic acid, or denosumab for 5 years for women with low BMD and osteoporosis. 37 Treatment requires individualization in women aged 65 or older, considering the risks versus the benefits, patient preferences, fracture risk profile, and costs. Menopausal estrogen therapy, combined estrogen and progestin therapy, and raloxifene are not widely recommended, and monitoring BMD for 5 years is controversial. 41 44 45

The selection of osteoporosis medications is often based on personal preference, convenience, and adherence to the dosing schedule. The ideal duration of the bisphosphonate treatment is controversial due to the uncertainty of atypical femoral fracture. However, most experts suggest a break after 5 years, especially for patients with no fragility fractures and bone density maintenance. 46 Although medications reduce the likelihood of fractures, they do not eliminate all risks, and complementary treatments such as physical exercise and physical therapy are equally important in osteoporosis management. 46

### Non-pharmacological Management and Exercise

The management of non-pharmacological osteoporosis encompasses adequate calcium and vitamin D intake, weight-bearing exercises, smoking cessation, limitation of alcohol/caffeine consumption, and fall prevention techniques. Vitamin D plays a crucial role in calcium absorption and bone health, and lower daily doses are recommended to reduce the risk of falls. 46 Physical therapy is essential to strengthen muscles, improve joint movements, and reduce sarcopenia, promoting a more stable gait. Implementing balanced exercise routines can significantly contribute to fall prevention, a common factor in fractures in osteoporotic patients.

### Fall Prevention

Falls in the elderly population result from a complex interaction between intrinsic and extrinsic factors, with environmental risks contributing to around 40% of falls. Factors such as socioeconomic deprivation increase fracture incidence; more than 90% of fractures result from low-energy falls, with significant mortality rates. 47 48 49 50 Aging, comorbidities, and premature hospital discharge increase mortality rates. Studies 2 3 4 17 48 indicate that half of falls occur while walking, often due to trips or slips, while environmental factors, such as uneven surfaces, wet floors, and loose objects, are frequent triggers. The increasing tendency for outdoor falls also relates to extrinsic factors.

Women who walk quickly and trip are more likely to fall forward, which may result in wrist, arm, and elbow fractures. In contrast, falls to the side can cause hip and spinal fractures. 16 17 18 19 Preventing fractures involves preserving well-paved streets, placing warning signs in areas with an elderly population, and investing in public health policies. Shoes with high and wide heels play a significant role in such falls, and the recommendation is to limit their use. 37 49 50 51 52 53 Since medications, in addition to vision and hearing issues, can affect balance, balance programs are beneficial to improve stability. Tai Chi Chuan is an excellent exercise to increase stability and prevent falls in the elderly. 5 37 42 43

In the Timed Up and Go test, 54 the patient gets up from a chair, walks 5 meters, returns, and sit downs without using their arms for support. As a prophylactic measure, crutches or walkers are recommended for patients unable to perform the test without support or at risk of falling. Furthermore, domestic violence can cause falls in the elderly, requiring compulsory notification when confirmed.

## Final Considerations

Osteoporosis increases the susceptibility to fractures and leads to substantial rates of morbidity and mortality in the elderly. Its diagnosis includes bone densitometry testing to assess BMD in the bones most likely to fracture, such as the lower spine (lumbar vertebrae), femoral neck, and forearm bones. Osteoporosis may be primary or secondary (resulting from several comorbidities, medications, or both).

Osteoporosis is often diagnosed after the patient's first clinical fracture. Most subjects with osteoporotic fractures require orthopedic surgical treatment. Osteoporosis is usually age-related and underdiagnosed because it remains asymptomatic (silent disease) for several years until the occurrence of a fracture. The FRAX calculates the 10-year probability of a severe osteoporotic fracture. It is necessary to develop public health policies focused on preventing secondary frailty and constantly invest in public health policies focused on providing social and economic benefits to the elderly.

Osteoporosis treatment is multidisciplinary and must include periodic assessment of the patient, prescription of new bone medications, and guidance on fall prevention. Bisphosphonates are the most common medications prescribed, but their prolonged use can cause atypical femoral fractures and patients should be informed about this potential risk.

**Table 2:** T-scores and diagnostic criteria of the World Health Organization for osteoporosis 2

T-score*	
> −1	Normal bone density
From −1 to −2.5	This score is a sign of osteopenia; bone density is lower than normal and can lead to osteoporosis.
< −2.5	This bone density indicates that the person is likely to have osteoporosis.

**Note:** *Reference values range depending on geographical location.
